# High expression of RRM2 mediated by non-coding RNAs correlates with poor prognosis and tumor immune infiltration of hepatocellular carcinoma

**DOI:** 10.3389/fmed.2022.833301

**Published:** 2022-07-14

**Authors:** Guochao Mao, Lan Li, Changyou Shan, Baobao Liang, Li Ma, Shuqun Zhang

**Affiliations:** ^1^Department of Oncology, The Second Affiliated Hospital of Xi’an Jiaotong University, Xi’an, China; ^2^Department of Breast Surgery, Shaanxi Provincial Cancer Hospital, Xi’an, China

**Keywords:** HCC, RRM2, ncRNAs, regulatory mechanism, immune cell infiltration

## Abstract

Hepatocellular carcinoma (HCC) is known to have a poor prognosis. Accumulating evidence indicates that RRM2 plays a critical role in the occurrence and progression of multiple human cancers. However, the knowledge about RRM2 in HCC is still insufficient, and further research is needed. Here, we first analyzed the expression and prognosis of RRM2 using TCGA and GTEx data, and found that RRM2 may play a potential carcinogenic role in HCC. Then, through a series of comprehensive analysis, including expression analysis, correlation analysis or survival analysis, non-coding RNAs (ncRNAs) that regulate RRM2 overexpression were identified. Finally, MIR4435-2HG/CYTOR were observed to be the most promising upstream lncRNAs for the miR-125b-5p/RRM2 axis in HCC. In addition, RRM2 expression was significantly positively related to immune cell infiltration, immune cell biomarker or immune checkpoint expression in HCC. Altogether, the upregulation of RRM2 mediated by ncRNAs correlates with poor prognosis and tumor immune infiltration of HCC.

## Introduction

Hepatocellular carcinoma (HCC) is a common cause of cancer-related deaths with increasing mortality worldwide, and has very poor prognosis with an incidence rate almost equal to the mortality rate (1, 2). Risk factors associated with the etiology of HCC include hepatitis type B and C, cirrhosis, alcoholism, diabetes, non-alcoholic fatty liver disease (NAFLD) and toxin exposure (3, 4). Despite these encouraging advancements in prevention, diagnosis, prognosis, and treatment, the options and outcome for this deadly cancer remain very limited. For example, sorafenib, a protein tyrosine kinase inhibitor for the treatment of HCC, which can only prolong the survival time by about 3 months. Thus, there are urgent calls for effective diagnostic and therapeutic regimens.

Ribonucleotide reductase M2 subunit (RRM2), a small subunit of the ribonucleotide reductase complex (5), is a rate-limiting enzyme responsible for DNA synthesis and DNA repair by producing dNTP (6). Ribonucleotide reductase (RNR) activity consists of two subunits (regulatory subunit RRM1 and catalytic subunit RRM2), which are coordinated with the process of cell cycle to maintain a fine balance between dNTP production and DNA replication (7). Unlike the tumor suppressor of RRM1 (8), RRM2 has carcinogenic activity and is widely involved in tumor growth, metastasis and drug resistance in different types of cancer (9–11). During the cell cycle, the expression of RRM2 depends on the cell cycle and reaches the highest level in S-phase (12). RRM2 is also related to poor prognosis and overexpressed in a variety of human cancers, such as breast cancer (13), lung cancer (14), colorectal cancer (15), glioma (16), renal cell carcinoma (17), and prostate cancer (10). RRM2 can be used as a prognostic biomarker to predict the survival and potential therapeutic target in these cancer patients. Growing evidence suggests that RRM2 may be a promising cancer therapeutic target. For instance, a RRM2 inhibitor, COH29, can inhibits the growth of gemcitabine resistant cancer cells (10). In addition, another RRM2 inhibitor, GW8510, has shown to inhibit the growth of breast cancer (18) and lung cancer (19). Although there are some reports of RRM2 regulation in lung and breast cancer, there is no comprehensive molecular understanding of how RRM2 is regulated by key upstream biological processes. Especially, very little is known about the regulatory mechanism and function of RRM2 in HCC. In addition, the correlation between RRM2 and immune infiltration in HCC remain unclear.

Here, we first investigated RRM2’s expression and survival analysis across multiple cancer types. Subsequently, the regulation mechanism of RRM2 related to non-coding RNA (ncRNA) involving small RNA (miRNA) and long non-coding RNA (lncRNA) was also discussed in HCC. Lastly, we identified the correlation between RRM2 expression and immune cell infiltration, biomarkers and immune checkpoints in HCC. In conclusion, our findings suggest that the upregulation of RRM2 mediated by ncRNAs correlates with poor prognosis and tumor immune infiltration of HCC patients.

## Results

### RRM2 expression analysis in pan-cancer

**FIGURE 1:**
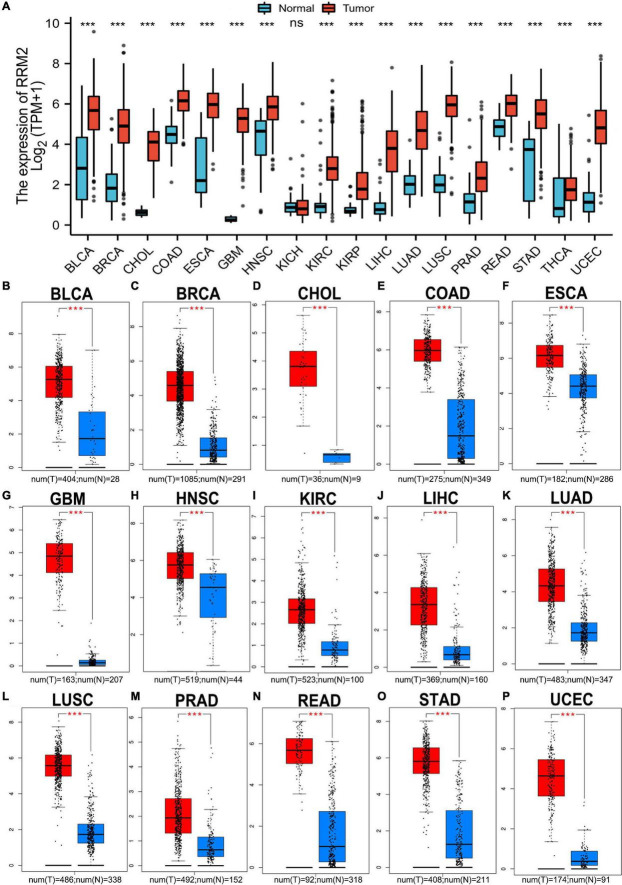
Pan-cancer analysis of RRM2 expression **(A)** RRM2 expression analysis across cancer types (18) using TCGA database. **(B–P)** RRM2 expression analysis using GEPIA database in BLCA **(B)**, BRCA **(C)**, CHOL **(D)**, COAD **(E)**, ESCA **(F)**, GBM **(G)**, HNSC **(H)**, KIRC **(I)**, LIHC **(J)**, LUAD **(K)**, LUSC **(L)**, PRAD **(M)**, READ **(N)**, STAD **(O)**, and UCEC **(P)**. TPM, transcripts per million; Red, tumor group; Blue, normal group; ns, no significance; ***p value < 0.001.

As an initial investigation of RRM2 in carcinogenesis, we evaluated RRM2 expression in 18 human cancer types. As shown in Figure 1A, we found that there was no significant change in RRM2 expression in KICH compared with normal tissues, but RRM2 expression was significantly increased in the remaining 17 cancer types, including BLCA, BRCA, CHOL, COAD, ESCA, GBM, HNSC, KIRC, KIRP, LIHC, LUAD, LUSC, PRAD, READ, STAD, THCA, and UCEC. Additionally, to further confirm the expression of RRM2 in multiple cancers, we carried out RRM2 expression analysis using GEPIA. As shown in Figures 1B–P, we observed consistent results of elevated RRM2 expression in BLCA, BRCA, CHOL, COAD, ESCA, GBM, HNSC, KIRC, LIHC, LUAD, LUSC, PRAD, READ, STAD, and UCEC. Date from the pan-cancer analysis revealed significant upregulation of RRM2 across a variety of cancer types, suggesting a specific function of RRM2 in these 15 types of cancer related to tumorigenesis.

### The prognostic value of RRM2 in human cancer

**FIGURE 2:**
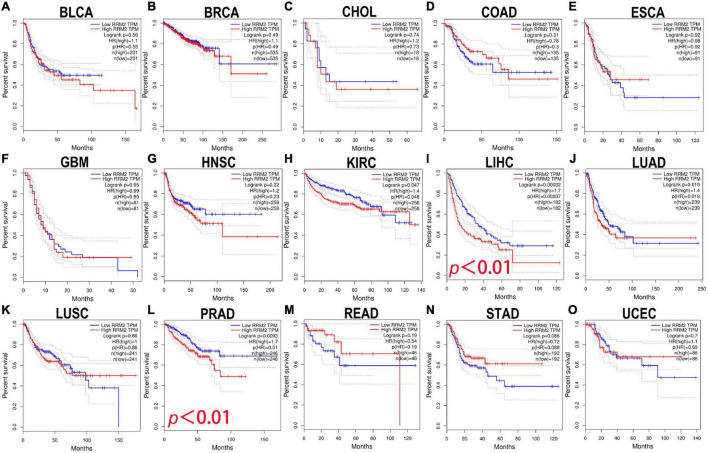
The prognostic analysis of RRM2 expression in disease-free survival (RFS) of various tumors using GEPIA. **(A–O)** The RFS curve of RRM2 in BLCA **(A)**, BRCA **(B)**, CHOL **(C)**, COAD **(D)**, ESCA **(E)**, GBM **(F)**, HNSC **(G)**, KIRC **(H)**, LIHC **(I)**, LUAD **(J)**, LUSC **(K)**, PRAD **(L)**, READ **(M)**, STAD **(N)**, and UCEC **(O)**.

**FIGURE 3:**
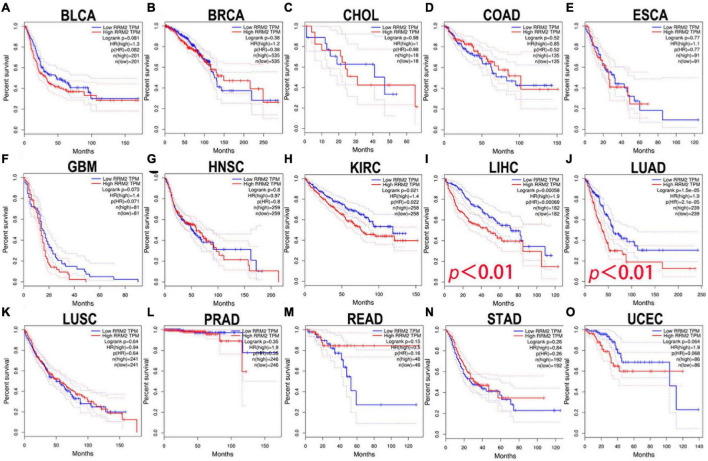
The prognostic analysis of RRM2 expression in overall survival (OS) of various tumors using GEPIA. **(A–O)** The OS curve of RRM2 in BLCA **(A)**, BRCA **(B)**, CHOL **(C)**, COAD **(D)**, ESCA **(E)**, GBM **(F)**, HNSC **(G)**, KIRC **(H)**, LIHC **(I)**, LUAD **(J)**, LUSC **(K)**, PRAD **(L)**, READ **(M)**, STAD **(N)**, and UCEC **(O)**.

Here, the GEPIA was used to explore the prognostic significance of RRM2 expression in various tumors, including BLCA, BRCA, CHOL, COAD, ESCA, GBM, HNSC, KIRC, LIHC, LUAD, LUSC, PRAD, READ, STAD, and UCEC. Study metrics included disease-free survival (RFS) and overall survival (OS). In RFS analysis, high expression of RRM2 in LIHC and PRAD was associated with poor prognosis (Figure 2). In OS analysis, high RRM2 expression in LIHC and LUAD predicts poor outcomes (Figure 3). The correlation between RRM2 and prognosis was not observed in other tumors. Finally, we conclude that RRM2 predicted poor prognosis in patients with HCC, indicating its unique prognostic biomarker in HCC patients.

### Prediction and analysis of upstream miRNAs of RRM2

**FIGURE 4:**
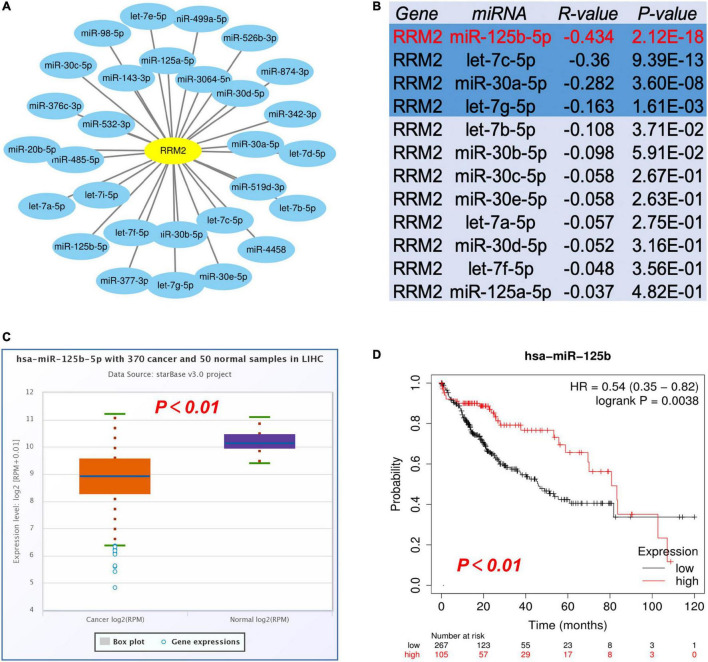
Identification of miR-125b-5p as a potential upstream regulatory miRNA of RRM2 in HCC. **(A)** The miRNA-RRM2 prediction network produced by Cytoscape. **(B)** The correlation between the predicted expression of some candidate miRNAs and RRM2 in HCC analyzed by starBase. **(C,D)** The expression and prognostic value of miR-125b-5p in HCC were detected by starBase and Kaplan Meier plotter.

The most widely known ncRNA system is post-transcriptional regulation of gene expression by miRNAs. We first performed prediction of the upstream miRNAs that might bind RRM2, and found 29 candidate miRNAs. We also used Cytoscape software based interaction maps to represent the miRNA-RRM2 regulatory network (Figure 4A). Next, based on the negative regulation mechanism of miRNA, RRM2 should be negatively related to miRNA. Therefore, we performed correlation analysis. As shown in Figure 4B, there was a statistically significant negative correlation between RRM2 and four miRNAs in HCC, including miR-125b-5p (*r* = −0.434, *p* = 2.12e-18), let-7c-5p (*r* = −0.36, *p* = 9.39e-13), miR-30a-5p (*r* = −0.282, *p* = 3.60e-8) and let-7g-5p (*r* = −0.163, *p* = 1.61e-03). The other miRNAs did not enter the next research step. Finally, we evaluated the expression and prognostic value of these four miRNAs in HCC. All four miRNAs were down regulated in HCC, only the expression of miR-125b-5p (*p* = 0.0038) was statistically correlated with the prognosis of HCC by Kaplan-Meier plotter analysis (Figures 4C,D and Supplementary Figure 1). Taken these together, these results indicated that miR-125b-5p may be the most potential upstream regulator of RRM2 in HCC.

### Prediction and analysis of upstream lncRNAs of miR-125b-5p

**FIGURE 5:**
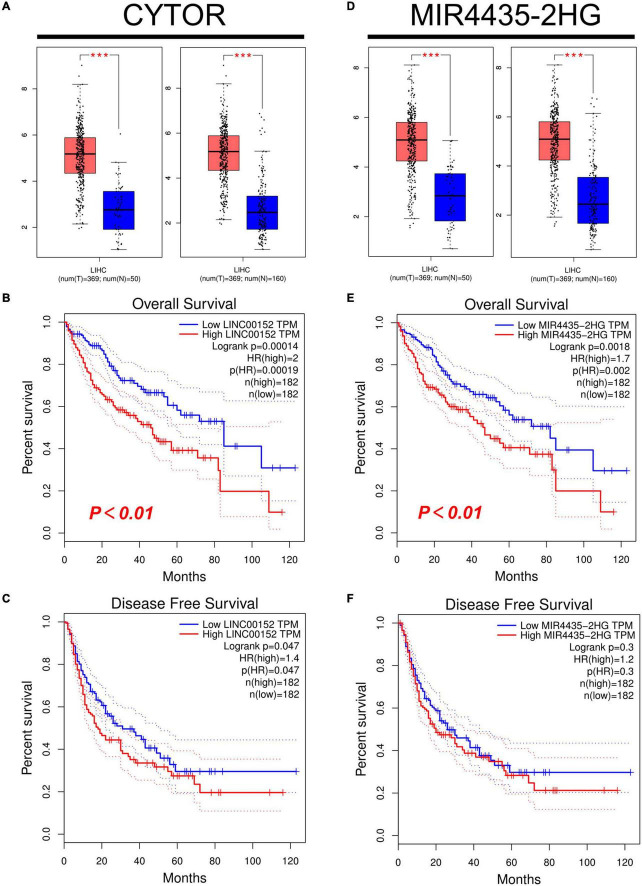
Expression and survival analysis of upstream lncRNAs of miR-125b-5p in HCC. **(A,D)** The expression of CYTOR **(A)** and MIR4435-2HG **(D)** in TCGA HCC compared with “TCGA normal” or “TCGA and GTEx normal” data. **(B,E**) The OS analysis for CYTOR **(B)** and MIR4435-2HG **(E)** in HCC. **(C,F)** The RFS for CYTOR **(C)** and TMPO-AS1 **(F)** in HCC, ***p value < 0.001.

Firstly, we used starBase to predict the upstream lncRNAs of miR-125b-5p in HCC. As a result, a total of 47 possible lncRNAs were included. The visualization of miR-125b-5p regulatory network was plotted with Cytoscape software (Supplementary Figure 2). Then, we used GEPIA to evaluate the expression of these lncRNAs in HCC. Among all 47 lncRNAs, only the expression of CYTOR and MIR4435-2HG increased significantly upregulated in HCC compared with normal samples (Figure 5A, *p* < 0.01, Figure 5D, *p* < 0.01). Finally, we evaluated the prognostic value of these two lncRNAs in HCC. Patients with higher expression of CYTOR and MIR4435-2HG showed worse OS rather than RFS in HCC (Figures 5B,C,E,F).

The competing endogenous RNA (ceRNA) hypothesis was presented to explain the relationship among various RNAs. According to the ceRNA hypothesis, there should be positive association between lncRNA expression and mRNA expression or negative correlation between lncRNA and miRNA. We then used the starBase database to analyze the correlation between lncRNA and RRM2 or lncRNA and miR-125b-5p in HCC. The results were in line with ceRNA theory (Table 1). As shown in Table 1, we found that lncRNAs (CYTOR and MIR4435-2HG) were negatively correlated with miR-125b-5p (*r* = −0.372, *p* = 1.35e-13; *r* = −0.307, *p* = 1.70e-09, respectively), but positively correlated with RRM2 (*r* = 0.342, *p* = 1.11e-11; *r* = 0.239, *p* = 3.08e-06, respectively). Combined with all the above analysis, it is finally concluded that lncRNA CYTOR and MIR4435-2HG may be the two most potential upstream regulators of the miR-125b-5p/RRM2 axis in HCC.

**TABLE 1:** Association between lncRNA and miR-125b-5p or lncRNA and RRM2 in HCC analyzed by starBase.

lncRNA	miRNA	*R*-value	*P*-value
CYTOR	miR-125b-5p	−0.372^a^	1.35e-13***
MIR4435-2HG	miR-125b-5p	−0.307^a^	1.70e-09***
lncRNA	mRNA	*R*-value	*P*-value
CYTOR	RRM2	0.342^a^	1.11e-11***
MIR4435-2HG	RRM2	0.239^a^	3.08e-06***

^a^means statistically significant.

***p value < 0.001.

### Correlation of RRM2 with immune cell infiltration in hepatocellular carcinoma

**FIGURE 6:**
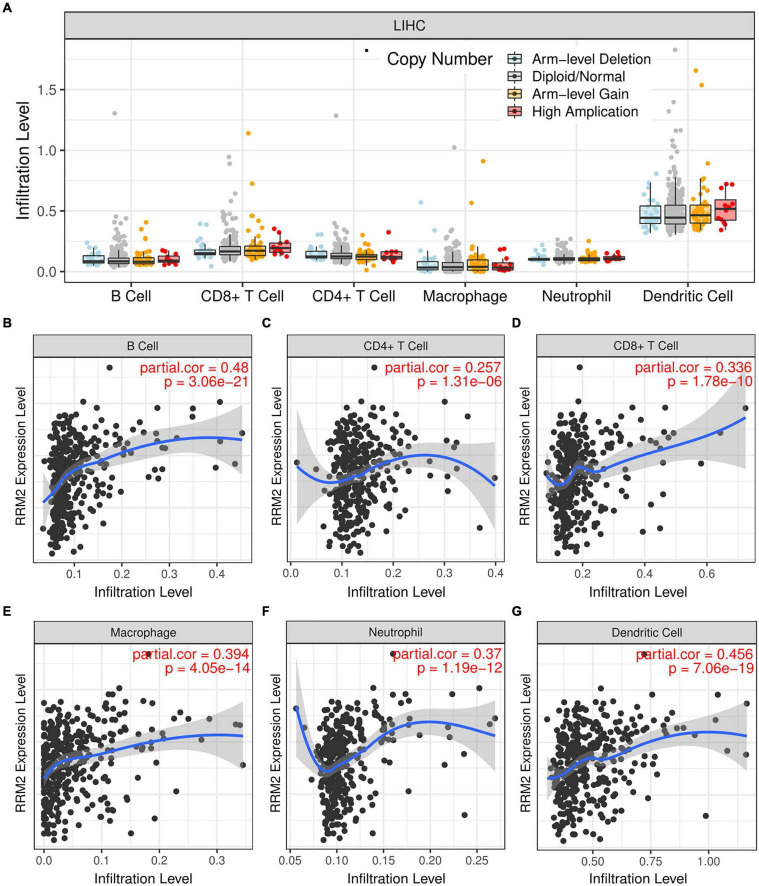
Correlation analysis between immune cell infiltration and RRM2 expression in HCC. **(A)** Effects of different RRM2 copy numbers on different immune cell infiltration in HCC. **(B–G)** Correlation between expression level of RRM2 and infiltration level of B cells **(B)**, CD4 + T cells **(C)**, CD8 + T cells **(D)**, macrophages **(E)**, neutrophils **(F)** or dendritic cells **(G)** in HCC.

To verify the correlation between RRM2 and immune cell infiltration, we tested whether RRM2-expressing cancers exhibited gene expression signatures of high immune infiltration. First, we observed that the level of immune cell infiltration did not change with the change of RRM2 copy number in HCC (Figure 6A). Correlation analysis can provide important clues to reveal the function and mechanism of RRM2. Therefore, we evaluated the correlation between RRM2 expression level and immune cell infiltration level. As presented in Figures 6B–G, our results showed that the expression of RRM2 was significantly positively correlated with all analyzed immune cells, including B cells (*r* = 0.48, *p* = 3.06e-21), CD8 + T cells (*r* = 0.336, *p* = 1.78e-10), CD4 + T cells (*r* = 0.257, *p* = 1.31e-06), macrophages (*r* = 0.394, *p* = 4.05e-14), neutrophils (*r* = 0.37, *p* = 1.19e-12) and dendritic cells (*r* = 0.456, *p* = 7.06e-19) in HCC.

### Association between RRM2 and immune cell biomarkers in hepatocellular carcinoma

In this part, we used the GEPIA to analyze the correlation between RRM2 and the expression of immune cell biomarkers in HCC. As presented in Table 2, RRM2 was significantly positively related to dendritic cell’s biomarkers (HLA-DPB1, HLA-DPA1, HLA-DRA, ITGAX, and NRP1), B cell’s biomarkers (CD79A and CD19), M2 macrophage’s biomarkers (MS4A4A and VSIG4), CD4 + T cell’s biomarker (CD4), CD8 + T cell’s biomarkers (CD8A and CD8B), neutrophil’s biomarkers (ITGAM) and M1 macrophage’s biomarkers (IRF5) in HCC. These results further support the positive correlation between RRM2 and immune cell infiltration in HCC.

**TABLE 2:** Association between RRM2 and immune cell biomarkers in HCC analyzed by GEPIA.

Immune cell	Biomarker	*R*-value	*P*-value
B cell	CD19	0.27^a^	8.9E-08***
	CD79A	0.16^a^	2.4E-03**
CD8 + T cell	CD8A	0.22^a^	1.8E-05***
	CD8B	0.21^a^	4.7E-05***
CD4 + T cell	CD4	0.32^a^	3.3E-10***
M1 macrophage	NOS2	0.057	2.7E-01
	IRF5	0.37^a^	1.1E-13***
	PTGS2	0.13	1.2E-02
M2 macrophage	CD163	0.12	1.7E-02*
	VSIG4	0.17^a^	1.1E-03**
	MS4A4A	0.19^a^	2.9E-04***
Neutrophil	CEACAM8	0.13	1.0E-02
	ITGAM	0.37^a^	1.1E-13***
	CCR7	0.12	2.6E-02
Dendritic cell	HLA-DPB1	0.22^a^	2.7E-05***
	HLA-DQB1	0.12	2.4E-02
	HLA-DRA	0.25^a^	1.1E-06***
	HLA-DPA1	0.22^a^	3.0E-05***
	CD1C	0.12	2.2E-02
	NRP1	0.18^a^	3.6E-04***
	ITGAX	0.37^a^	2.5E-13***

^a^means statistically significant.

*p-value < 0.05; **p-value < 0.01; ***p-value < 0.001.

### Expression correlation of RRM2 and immune checkpoints in hepatocellular carcinoma

**FIGURE 7:**
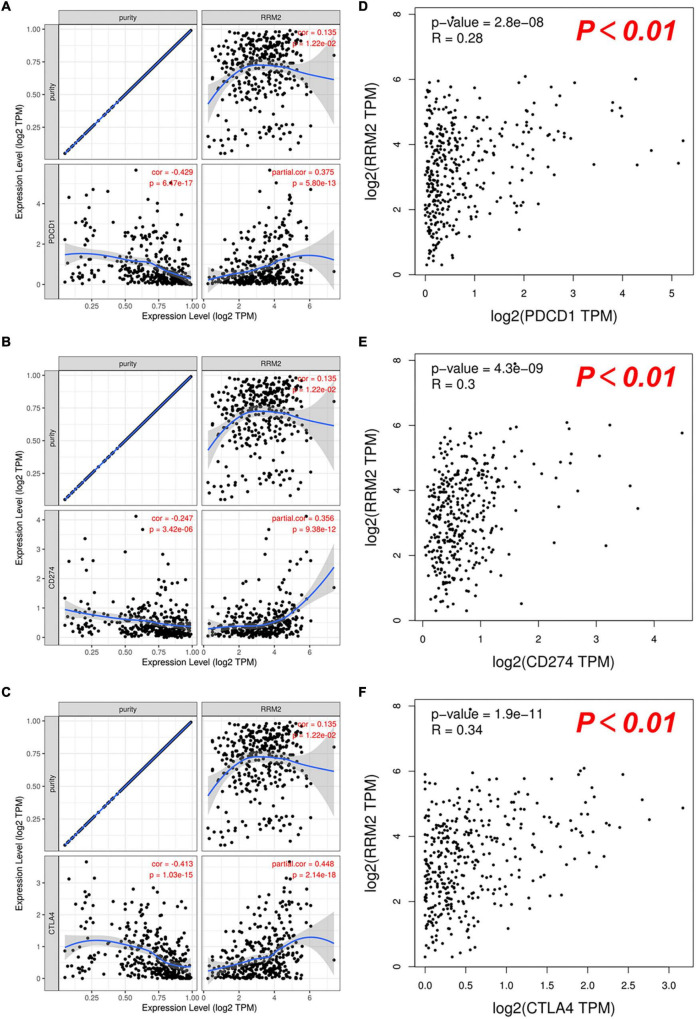
Correlation between RRM2 expression and PD-1, PD-L1 or CTLA-4 expression in HCC. **(A–C)** Spearman correlation of RRM2 with expression of PD-1 **(A)**, PD-L1 **(B)**, or CTLA-4 **(C)** in HCC determined by TIMER. **(D–F)** The correlation of RRM2 with expression of PD-1 **(D)**, PD-L1 **(E)**, or CTLA-4 **(F)** in HCC determined by GEPIA.

PD1, PD-L1, and CTLA-4 were found to be immune checkpoints responsible for tumor immune escape and key targets of immunotherapy. We further evaluated the relationship between RRM2 and PD1, PD-L1 or CTLA-4 in HCC using TIMER and GEPIA. As presented in Figure 7, the analysis results of the two databases were consistent, showing that the expression of RRM2 in HCC was significantly positively related to the expression of PD1, PD-L1, and CTLA-4 in HCC. Altogether, our findings suggest that tumor immune escape may be involved in hepatocarcinogenesis mediated by RRM2.

## Discussion

Hepatocellular carcinoma is known to have a poor prognosis. Various mechanisms have proved to be involved in the occurrence of HCC. Clarifying the molecular mechanism of HCC is helpful for the development of therapeutic targets, or the discovery of new and ideal prognostic clinical markers. A growing body of evidence shows that RRM2 plays a key role in the occurrence and progression of a variety of human cancers, including HCC. Growing evidence points to the RRM2 as a key role in the occurrence and progression of a variety of human cancers, including HCC. Yet, the knowledge about RRM2 in HCC is still insufficient, and further research is needed.

In this study, we first used TCGA to perform a pan-cancer expression analysis of RRM2, and then further used GEPIA to confirm the expression of RRM2. Our results showed that RRM2 expression is elevated in most cancer types (including HCC), and this high expression indicates a poor prognosis in HCC patients. Studies have found that RRM2 showed specifically elevated levels in HCC and inhibits ferroptosis by stimulating glutathione synthesis via glutathione synthetase, thus participating in the occurrence of HCC (20). Pei et al. (21) have proved that sorafenib can inhibit RRM2 and exert anticancer activity. Together with our results, these reports show the carcinogenic effect of RRM2 in HCC.

A growing number of ncRNAs are being considered as regulators of various cellular processes, including cancer, and play a regulatory role through ceRNA mechanism (22–26). Given the involvement of ncRNAs in carcinogenesis and tumor heterogeneity, a deeper understanding of its molecular function is imperative. In this study, we searched for upstream miRNAs that might bind to RRM2 using starBase software. Through analysis, we finally obtained 29 candidate miRNAs, most of which were found to play a role as tumor suppressor in HCC. For instance, let-7c-5p increased the antitumor effect of 5-FU by inhibiting the expression of ABCC5/MRP5 (27). miR-30a-5p can reverse the resistance of sorafenib in HCC cells by directly targeting CLCF1, thereby exerting an anti-tumor effect (28). Let-7b-5p can slow down G2/M cell cycle transition by directly target CDC25B/CDK1 axis, and inhibit HCC cell metastasis and EMT progression by targeting HMGA2 (29). MiR-30b-5p acts as a tumor suppressor and inhibits cell proliferation and cell cycle in HCC by targeting DNMT3A and USP37 (30). Combined with our results, we believe that miR-125b-5p may be the most potential upstream regulator of RRM2 in HCC.

According to the ceRNA hypothesis, the potential upstream lncRNAs of miR-125b-5p/RRM2 axis should be carcinogenic in HCC. Subsequently, we predicted the upstream lncRNAs of this axis and found 47 possible lncRNAs. Through a series of comprehensive analysis, two most potential candidate up-regulated lncRNAs were finally determined, namely CYTOR and MIR4435-2HG. Combined with literature reports, these two lncRNAs play a role as oncogenes in a variety of malignant tumors, including HCC. For example, CYTOR promotes the proliferation and cell cycle of hepatoma cells and inhibits the apoptosis of hepatoma cells through miR-125b-5p/KIAA1522 axis (31). MIR4435-2HG promotes HCC proliferation and metastasis through the miR-22-3p/YWHAZ axis (32) and miRNA-487a (33). Therefore, we identified lncRNAs (CYTOR and MIR4435-2HG) may be the two most potential upstream regulators of the miR-125b-5p/RRM2 axis in HCC.

Infiltration of solid tumors by immune cells is a hallmark of cancer and plays a key role in tumor progression (34). The tumor microenvironment may reprogram tumor-infiltrating immune cells to obtain tumor-promoting functions that promote tumor growth (35, 36). In addition to the characteristics of tumor autonomy, the pattern of invasive immune cell types is also related to tumor progression and patient prognosis (37, 38). This study showed that RRM2 was significantly positively related to most immune cells in HCC. In addition, RRM2 was also significantly positively associated with these biomarkers infiltrating immune cells. These results suggest that tumor immune infiltration could play a vital role in RRM2-mediated development of HCC. Based on our results, it seems that immune-infiltrating cells are negatively correlated with the prognosis of HCC patients, but we did not study the prognostic correlation between immune-infiltrating cells and HCC patients. This also reflects the complex role of immune infiltrating cells in HCC, which need further exploration.

The expression of immune checkpoint molecules in tumor tissues is very important for the efficacy of immune checkpoint blockade drugs (39–42). Immune checkpoint receptors can either inhibit or enhance immune response pathways (43). Therefore, we evaluated the correlation between RRM2 and immune checkpoints. Our findings showed that the high expression of RRM2 in HCC was closely related to PD1, PD-L1, and CTLA-4, suggesting that tumor immune escape may be involved in RRM2-mediated hepatocarcinogenesis, and RRM2 may be a synergistic therapeutic target related to immunotherapy in HCC.

**FIGURE 8:**
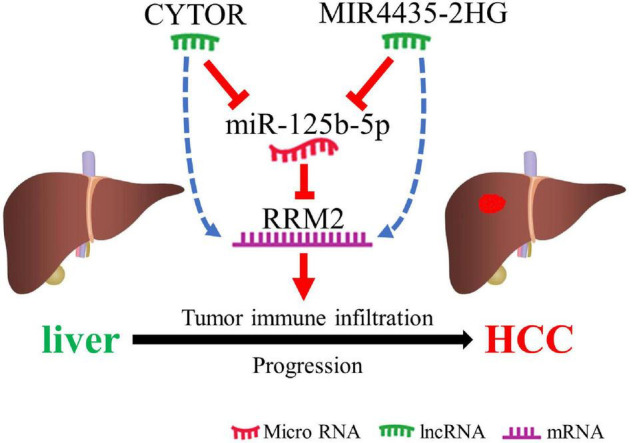
The schematic diagram of MIR4435-2HG/CYTOR – miR-125b-5p – RRM2 in hepatocarcinogenesis.

In conclusion, we clarified that RRM2 is up-regulated in a variety of human cancers (including HCC), and is positively related to the poor prognosis of HCC. We suggested that lncRNAs (CYTOR and MIR4435-2HG) may be the two most potential upstream regulators of the miR-125b-5p/RRM2 axis in HCC (Figure 8). In addition, our current results also suggest that RRM2 may play a carcinogenic role by increasing immune cell infiltration and immune checkpoint expression. However, these findings still need to be further proved with more benchwork studies and clinical studies.

## Materials and methods

### mRNA expression analysis

All available mRNA expression data was downloaded from the following projects: TCGA-ALL. Fragments per kilobase per million (FPKM) were subsequently transformed to transcripts per million (TPM). The date of RRM2 expression in 18 cancer types (BRCA, BLCA, COAD, CHOL, ESCA, HNSC, GBM, KICH, KIRP, KIRC, LIHC, LUSC, LUAD, PRAD, STAD, READ, UCEC, and THCA) were performed using a Mann-Whitney *U* test with the R package ggplot2. This study complies with the publication guidelines and access rules of TCGA.

### GEPIA

GEPIA is an RNA sequencing database derived from analysis of 9,736 tumors and 8,587 healthy samples from TCGA and GTEx (44). RRM2 and lncRNA expression profiles in patients across cancers were matched with TCGA normal and GTEx data, and analyzed using GEPIA. GEPIA was also used to perform survival analysis of RRM2 and lncRNAs in HCC with log-rank test, including OS and RFS. The expression correlation between RRM2 and immune checkpoints in HCC was also determined using Spearman test within GEPIA. For correlation analysis, | R| > 0.1 and *p* < 0.01 were set as the criteria for identifying significant interactive pairs.

### Identification of candidate novel miRNA

Candidate novel miRNAs to regulate RRM2 expression were predicted using following miRNA target prediction databases: PITA, miRmap, RNA22, microT, PicTar, TargetScan, and miRanda. Only miRNAs that were predicted by at least two out of the seven databases and were then retained for subsequent steps. Finally, the filtered ones were marked as “candidate” miRNAs of RRM2.

### Upstream lncRNAs prediction

starBase collects miRNA-ceRNA, miRNA-ncRNA, and protein-RNA interaction networks from large-scale CLIP-Seq data, and is usually used to study lncRNA-miRNA-mRNA networks (45). The starBase 3.0 software was used to identify potential complementary lncRNA binding partners for miR-125b-5p. Then, the starBase was employed to study the expression correlation between lncRNA and miR-125b-5p or lncRNA and RRM2 in HCC.

### Kaplan-Meier plotter

Kaplan-Meier plotter^1^, an *in silico* online tool was used to predict survival of cancer types patients depending on the different gene expression (46). Survival analysis for candidate ncRNAs (lncRNAs and miRNAs) in HCC was achieved using Kaplan-Meier plotter.

### TIMER

TIMER database is a web tool for the comprehensive analysis and visualization of immune cells infiltration in tumors. It provides the infiltration of six kinds of immune cells (CD4 + T cells, CD8 + T cells, B cells, neutrophils, macrophages and dendritic cells). The correlation between RRM2 expression and tumor-infiltrating or immune checkpoint expression in HCC was analyzed and visualized using Spearman’s rho value within TIMER.

### Statistical analysis

The statistical analysis was automatically calculated by the online database mentioned above or via the R software (v.4.1.3). A *p*-value < 0.01 or log-rank *p*-value < 0.01 was considered to be statistically significant.

## Data Availability Statement

The datasets presented in this study can be found in online repositories. The names of the repository/repositories and accession number(s) can be found in the article/Supplementary Material.

## Author contributions

GM and SZ conceived and designed the research. CS performed the data collection, bioinformatics analysis, and wrote the initial manuscript. BL and LM analyzed the data and generated the figures and tables. GM and LL conceptualized and revised the manuscript. All authors approved the final manuscript.

## Conflict of Interest

The authors declare that the research was conducted in the absence of any commercial or financial relationships that could be construed as a potential conflict of interest.

## Publisher’s Note

All claims expressed in this article are solely those of the authors and do not necessarily represent those of their affiliated organizations, or those of the publisher, the editors and the reviewers. Any product that may be evaluated in this article, or claim that may be made by its manufacturer, is not guaranteed or endorsed by the publisher.

## Footnotes

1
http://kmplot.com/analysis/

## Abbreviations

HCC: Hepatocellular carcinoma
RRM2: Ribonucleotide reductase M2 subunit
TCGA: the cancer genome atlas
ncRNAs: non-coding RNAs
NAFLDL: non-alcoholic fatty liver disease
miRNA: microRNA
lncRNA: long non-coding RNA
ceRNA: competing endogenous RNA
OS: overall survival
RFS: disease-free survival
BLCA: bladder urothelial carcinoma
BRCA: breast invasive carcinoma
CHOL: cholangiocarcinoma
COAD: colon adenocarcinoma
ESCA: esophageal carcinoma
GBM: glioblastoma multiforme
HNSC: head and neck squamous cell carcinoma
KICH: kidney chromophobe
KIRC: kidney renal clear cell carcinoma
KIRP: kidney renal papillary cell carcinoma
LIHC: liver hepatocellular carcinoma
LUAD: lung adenocarcinoma
LUSC: lung squamous cell carcinoma
PRAD: prostate adenocarcinoma
READ: rectum adenocarcinoma
STAD: stomach adenocarcinoma
THCA: thyroid carcinoma
UCEC: uterine corpus endometrial carcinoma.

## References

[1] Mcglynn KA Petrick JL El-Serag HB. Epidemiology of hepatocellular carcinoma. *Hepatology.* (2021) 73:4–13. 10.1002/hep.31288 PMC7577946 32319693

[2] Sung H Ferlay J Siegel RL Laversanne M Soerjomataram I Jemal A Global cancer statistics 2020: GLOBOCAN estimates of incidence and mortality worldwide for 36 cancers in 185 countries. *CA Cancer J Clin.* (2021) 71:209–49. 33538338 10.3322/caac.21660

[3] Wei L Lee D Law CT Zhang MS Shen J Chin DW Genome-wide CRISPR/Cas9 library screening identified PHGDH as a critical driver for Sorafenib resistance in HCC. *Nat Commun.* (2019) 10:4681. 10.1038/s41467-019-12606-7 31615983 PMC6794322

[4] Kuznietsova H Dziubenko N Hurmach V Chereschuk I Motuziuk O Ogloblya O Water-soluble pristine C_60_ fullerenes inhibit liver fibrotic alteration and prevent liver cirrhosis in rats. *Oxid Med Cell Longev.* (2020) 2020:8061246. 10.1155/2020/8061246 32148657 PMC7044474

[5] Xiong W Zhang B Yu H Zhu L Yi L Jin X. RRM2 regulates sensitivity to sunitinib and PD-1 blockade in renal cancer by stabilizing ANXA1 and activating the AKT pathway. *Adv Sci.* (2021) 8:e2100881. 10.1002/advs.202100881 34319001 PMC8456228

[6] D’angiolella V Donato V Forrester FM Jeong YT Pellacani C Kudo Y Cyclin F-mediated degradation of ribonucleotide reductase M2 controls genome integrity and DNA repair. *Cell.* (2012) 149:1023–34. 10.1016/j.cell.2012.03.043 22632967 PMC3616325

[7] Nordlund P Reichard P. Ribonucleotide reductases. *Annu Rev Biochem.* (2006) 75:681–706. 16756507 10.1146/annurev.biochem.75.103004.142443

[8] Shu Z Li Z Huang H Chen Y Fan J Yu L Cell-cycle-dependent phosphorylation of RRM1 ensures efficient DNA replication and regulates cancer vulnerability to ATR inhibition. *Oncogene.* (2020) 39:5721–33. 10.1038/s41388-020-01403-y 32712628

[9] Chen CW Li Y Hu S Zhou W Meng Y Li Z DHS (trans-4,4’-dihydroxystilbene) suppresses DNA replication and tumor growth by inhibiting RRM2 (ribonucleotide reductase regulatory subunit M2). *Oncogene.* (2019) 38:2364–79. 10.1038/s41388-018-0584-6 30518875 PMC6705423

[10] Mazzu YZ Armenia J Chakraborty G Yoshikawa Y Coggins SA Nandakumar S A novel mechanism driving poor-prognosis prostate cancer: overexpression of the DNA repair gene, ribonucleotide reductase small subunit M2 (RRM2). *Clin Cancer Res.* (2019) 25:4480–92. 10.1158/1078-0432.CCR-18-4046 30996073 PMC6820162

[11] Yang Y Li S Cao J Li Y Hu H Wu Z. RRM2 regulated by LINC00667/miR-143-3p signal is responsible for non-small cell lung cancer cell progression. *Onco Targets Ther.* (2019) 12:9927–39. 10.2147/OTT.S221339 31819489 PMC6876211

[12] Zheng S Wang X Weng YH Jin X Ji JL Guo L siRNA knockdown of RRM2 effectively suppressed pancreatic tumor growth alone or synergistically with doxorubicin. *Mol Ther Nucleic Acids.* (2018) 12:805–16. 30153565 10.1016/j.omtn.2018.08.003 PMC6118156

[13] Li S Mai H Zhu Y Li G Sun J Li G MicroRNA-4500 inhibits migration, invasion, and angiogenesis of breast cancer cells via RRM2-dependent MAPK signaling pathway. *Mol Ther Nucleic Acids.* (2020) 21:278–89. 10.1016/j.omtn.2020.04.018 32615527 PMC7330432

[14] Gandhi M Groß M Holler JM Coggins SA Patil N Leupold JH The lncRNA lincNMR regulates nucleotide metabolism via a YBX1 - RRM2 axis in cancer. *Nat Commun.* (2020) 11:3214. 10.1038/s41467-020-17007-9 32587247 PMC7316977

[15] Liu Q Guo L Qi H Lou M Wang R Hai B A MYBL2 complex for RRM2 transactivation and the synthetic effect of MYBL2 knockdown with WEE1 inhibition against colorectal cancer. *Cell Death Dis.* (2021) 12:683. 10.1038/s41419-021-03969-1 34234118 PMC8263627

[16] Sun H Yang B Zhang H Song J Zhang Y Xing J RRM2 is a potential prognostic biomarker with functional significance in glioma. *Int J Biol Sci.* (2019) 15:533–43. 10.7150/ijbs.30114 30745840 PMC6367584

[17] Osako Y Yoshino H Sakaguchi T Sugita S Yonemori M Nakagawa M Potential tumor-suppressive role of microRNA-99a-3p in sunitinib-resistant renal cell carcinoma cells through the regulation of RRM2. *Int J Oncol.* (2019) 54:1759–70. 10.3892/ijo.2019.4736 30816432

[18] Li ZN Shu Y Chen CG Li XQ Li MY Zhao XH Acquired tamoxifen resistance is surmounted by GW8510 through ribonucleotide reductase M2 downregulation-mediated autophagy induction. *Biochem Biophys Res Commun.* (2020) 528:554–60. 10.1016/j.bbrc.2020.05.149 32505349

[19] Chen P Wu JN Shu Y Jiang HG Zhao XH Qian H Gemcitabine resistance mediated by ribonucleotide reductase M2 in lung squamous cell carcinoma is reversed by GW8510 through autophagy induction. *Clin Sci.* (2018) 132:1417–33. 10.1042/CS20180010 29853661

[20] Yang Y Lin J Guo S Xue X Wang Y Qiu S RRM2 protects against ferroptosis and is a tumor biomarker for liver cancer. *Cancer Cell Int.* (2020) 20:587. 10.1186/s12935-020-01689-8 33372599 PMC7720568

[21] Yang PM Lin LS Liu TP. Sorafenib inhibits ribonucleotide reductase regulatory subunit M2 (RRM2) in hepatocellular carcinoma cells. *Biomolecules.* (2020) 10:117. 10.3390/biom10010117 31936661 PMC7022495

[22] Chan JJ Tay Y. Noncoding RNA:RNA regulatory networks in cancer. *Int J Mol Sci.* (2018) 19:1310. 10.3390/ijms19051310 PMC5983611 29702599

[23] Smillie CL Sirey T Ponting CP. Complexities of post-transcriptional regulation and the modeling of ceRNA crosstalk. *Crit Rev Biochem Mol Biol.* (2018) 53:231–45. 29569941 10.1080/10409238.2018.1447542 PMC5935048

[24] Yang XZ Cheng TT He QJ Lei ZY Chi J Tang Z LINC01133 as ceRNA inhibits gastric cancer progression by sponging miR-106a-3p to regulate APC expression and the Wnt/β-catenin pathway. *Mol Cancer.* (2018) 17:126. 10.1186/s12943-018-0874-1 30134915 PMC6106894

[25] Chen J Yu Y Li H Hu Q Chen X He Y Long non-coding RNA PVT1 promotes tumor progression by regulating the miR-143/HK2 axis in gallbladder cancer. *Mol Cancer.* (2019) 18:33. 10.1186/s12943-019-0947-9 30825877 PMC6397746

[26] Sang Y Chen B Song X Li Y Liang Y Han D circRNA_0025202 regulates tamoxifen sensitivity and tumor progression via regulating the miR-182-5p/FOXO3a axis in breast cancer. *Mol Ther.* (2019) 27:1638–52. 31153828 10.1016/j.ymthe.2019.05.011 PMC6731174

[27] Jilek JL Tu MJ Zhang C Yu AM. Pharmacokinetic and pharmacodynamic factors contribute to synergism between Let-7c-5p and 5-fluorouracil in inhibiting hepatocellular carcinoma cell viability. *Drug Metab Dispos.* (2020) 48:1257–63. 10.1124/dmd.120.000207 33051247 PMC7684025

[28] Wei H Yan S Hui Y Liu Y Guo H Li Q CircFAT1 promotes hepatocellular carcinoma progression via miR-30a-5p/REEP3 pathway. *J Cell Mol Med.* (2020) 24:14561–70. 10.1111/jcmm.16085 33179443 PMC7754024

[29] Li S Peng F Ning Y Jiang P Peng J Ding X SNHG16 as the miRNA let-7b-5p sponge facilitates the G2/M and epithelial-mesenchymal transition by regulating CDC25B and HMGA2 expression in hepatocellular carcinoma. *J Cell Biochem.* (2020) 121:2543–58. 10.1002/jcb.29477 31696971

[30] Qin X Chen J Wu L Liu Z. MiR-30b-5p acts as a tumor suppressor, repressing cell proliferation and cell cycle in human hepatocellular carcinoma. *Biomed Pharmacother.* (2017) 89:742–50. 10.1016/j.biopha.2017.02.062 28273636

[31] Hu B Yang XB Yang X Sang XT. LncRNA CYTOR affects the proliferation, cell cycle and apoptosis of hepatocellular carcinoma cells by regulating the miR-125b-5p/KIAA1522 axis. *Aging.* (2020) 13:2626–39. 10.18632/aging.202306 33318318 PMC7880333

[32] Shen X Ding Y Lu F Yuan H Luan W. Long noncoding RNA MIR4435-2HG promotes hepatocellular carcinoma proliferation and metastasis through the miR-22-3p/YWHAZ axis. *Am J Transl Res.* (2020) 12:6381–94. 33194037 PMC7653602

[33] Kong Q Liang C Jin Y Pan Y Tong D Kong Q The lncRNA MIR4435-2HG is upregulated in hepatocellular carcinoma and promotes cancer cell proliferation by upregulating miRNA-487a. *Cell Mol Biol Lett.* (2019) 24:26. 10.1186/s11658-019-0148-y 30988676 PMC6449898

[34] Hanahan D Weinberg RA. Hallmarks of cancer: the next generation. *Cell.* (2011) 144:646–74. 21376230 10.1016/j.cell.2011.02.013

[35] Coussens LM Zitvogel L Palucka AK. Neutralizing tumor-promoting chronic inflammation: a magic bullet? *Science.* (2013) 339:286–91. 10.1126/science.1232227 23329041 PMC3591506

[36] Kalafati L Kourtzelis I Schulte-Schrepping J Li X Hatzioannou A Grinenko T Innate immune training of granulopoiesis promotes anti-tumor activity. *Cell.* (2020) 183:771–85.e12. 10.1016/j.cell.2020.09.058 33125892 PMC7599076

[37] Newman AM Liu CL Green MR Gentles AJ Feng W Xu Y Robust enumeration of cell subsets from tissue expression profiles. *Nat Methods.* (2015) 12:453–7. 25822800 10.1038/nmeth.3337 PMC4739640

[38] Stewart PA Welsh EA Slebos RJC Fang B Izumi V Chambers M Proteogenomic landscape of squamous cell lung cancer. *Nat Commun.* (2019) 10:3578. 10.1038/s41467-019-11452-x PMC6687710 31395880

[39] Schreiber RD Old LJ Smyth MJ. Cancer immunoediting: integrating immunity’s roles in cancer suppression and promotion. *Science.* (2011) 331:1565–70. 10.1126/science.1203486 21436444

[40] Kythreotou A Siddique A Mauri FA Bower M Pinato DJ. PD-L1. *J Clin Pathol.* (2018) 71:189–94. 29097600 10.1136/jclinpath-2017-204853

[41] Wang SS Liu W Ly D Xu H Qu L Zhang L. Tumor-infiltrating B cells: their role and application in anti-tumor immunity in lung cancer. *Cell Mol Immunol.* (2019) 16:6–18. 10.1038/s41423-018-0027-x 29628498 PMC6318290

[42] Petitprez F Meylan M De Reyniès A Sautès-Fridman C Fridman WH. The tumor microenvironment in the response to immune checkpoint blockade therapies. *Front Immunol.* (2020) 11:784. 10.3389/fimmu.2020.00784 32457745 PMC7221158

[43] Sharma P Allison JP. The future of immune checkpoint therapy. *Science.* (2015) 348:56–61. 25838373 10.1126/science.aaa8172

[44] Tang Z Li C Kang B Gao G Li C Zhang Z. GEPIA: a web server for cancer and normal gene expression profiling and interactive analyses. *Nucleic Acids Res.* (2017) 45:W98–102. 28407145 10.1093/nar/gkx247 PMC5570223

[45] Li JH Liu S Zhou H Qu LH Yang JH. starBase v2.0: decoding miRNA-ceRNA, miRNA-ncRNA and protein-RNA interaction networks from large-scale CLIP-Seq data. *Nucleic Acids Res.* (2014) 42:D92–7. 10.1093/nar/gkt1248 24297251 PMC3964941

[46] Menyhárt O Nagy Á Győrffy B. Determining consistent prognostic biomarkers of overall survival and vascular invasion in hepatocellular carcinoma. *R Soc Open Sci.* (2018) 5:181006. 10.1098/rsos.181006 PMC6304123 30662724

