# Supplementary material for: High expression of RRM2 mediated by non-coding RNAs correlates with poor prognosis and tumor immune infiltration of hepatocellular carcinoma
Source: Front Med (Lausanne). 2022 Jul 14;9:833301. doi: 10.3389/fmed.2022.833301 (PMC9330188; doi:10.3389/fmed.2022.833301)
Supplement: Supplementary file 1 [file Data_Sheet_1.docx]

Figure 1A

**Source data**: See the attachment named "HTseq FPKM";

**Statistical programs**: We use R (version 3.6.3) for statistical analysis and visualization, where the R package, mainly ggplot2 [version 3.3.3] is used to visualize the data results. Differential analysis of RRM2 expression in tumor and normal was performed using the Mann-Whitney U test. We have attached R code was attached at the end of the text.

Figure 1A-1P, Figure 2-6, Table1 & 2

**Source data** & **Statistical programs**: The websites provide a visual and friendly operation interface. Users can directly input the target gene to obtain the visual results. We used the following database website:

Fig 1A-1P: http://gepia.cancer-pku.cn/

Fig 2&3: http://kmplot.com/analysis/

Fig 4: http://starbase.sysu.edu.cn/ & http://kmplot.com/analysis/

Fig 5: http://gepia.cancer-pku.cn/

Fig 6: https://cistrome.shinyapps.io/timer/

Table 1: http://starbase.sysu.edu.cn/

Table 2: http://gepia.cancer-pku.cn/

Fig S1: http://starbase.sysu.edu.cn/ & http://kmplot.com/analysis/

Fig S2: http://starbase.sysu.edu.cn/

**Others**

Please note that our entire study is based on the analysis of public databases and the manuscript does not contain information about any original gels, microscopy images or flow cytometry data.

library(tidyverse)

library(ggplot2)

library(reshape2)

library(car)

library(rstatix)

set.seed(100)

data <- data.frame(x = rnorm(100, 2, 1), y = rnorm(100, 1, 1))

data2 <- melt(data)

data3 <- lapply(data, function(x) get_summary_stats(data.frame(x)))

data3

# $x

# # A tibble: 1 x 13

# variable n min max median q1 q3 iqr mad mean sd se ci

# <chr> <dbl> <dbl> <dbl> <dbl> <dbl> <dbl> <dbl> <dbl> <dbl> <dbl> <dbl> <dbl>

# 1 x 100 -0.272 4.58 1.94 1.39 2.66 1.26 0.974 2.00 1.02 0.102 0.203

#

# $y

# # A tibble: 1 x 13

# variable n min max median q1 q3 iqr mad mean sd se ci

# <chr> <dbl> <dbl> <dbl> <dbl> <dbl> <dbl> <dbl> <dbl> <dbl> <dbl> <dbl> <dbl>

# 1 x 100 -1.14 3.17 0.927 0.568 1.45 0.878 0.648 1.01 0.796 0.08 0.158

data3 <- rbind(data3[[1]], data3[[2]])

data3[1] <- c("x", "y")

## Shapiro-Wilk normality test

lapply(data, function(x) shapiro.test(x))

# $x

#

# Shapiro-Wilk normality test

#

# data: x

# W = 0.98836, p-value = 0.535

#

#

# $y

#

# Shapiro-Wilk normality test

#

# data: x

# W = 0.98532, p-value = 0.3348

## Levene's Test

leveneTest(value~variable, data = data2)

# Levene's Test for Homogeneity of Variance (center = median)

# Df F value Pr(>F)

# group 1 4.4476 0.03621 *

# 198

# ---

# Signif. codes: 0 â€˜***â€™ 0.001 â€˜**â€™ 0.01 â€˜*â€™ 0.05 â€˜.â€™ 0.1 â€˜ â€™ 1

t.test(value~variable, data = data2, var.equal = T)

# Two Sample t-test

#

# data: value by variable

# t = 7.6613, df = 198, p-value = 8.012e-13

# alternative hypothesis: true difference in means is not equal to 0

# 95 percent confidence interval:

# 0.7364913 1.2470521

# sample estimates:

# mean in group x mean in group y

# 2.002913 1.011141

t.test(value~variable, data = data2, var.equal = F)

# Welch Two Sample t-test

#

# data: value by variable

# t = 7.6613, df = 186.92, p-value = 9.657e-13

# alternative hypothesis: true difference in means is not equal to 0

# 95 percent confidence interval:

# 0.7363983 1.2471452

# sample estimates:

# mean in group x mean in group y

# 2.002913 1.011141

wilcox.test(value~variable, data = data2)

# Wilcoxon rank sum test with continuity correction

#

# data: value by variable

# W = 7844, p-value = 3.711e-12

# alternative hypothesis: true location shift is not equal to 0

summary(aov(value~variable, data = data2))

# Df Sum Sq Mean Sq F value Pr(>F)

# variable 1 49.18 49.18 58.7 8.01e-13 ***

# Residuals 198 165.90 0.84

# ---

# Signif. codes: 0 â€˜***â€™ 0.001 â€˜**â€™ 0.01 â€˜*â€™ 0.05 â€˜.â€™ 0.1 â€˜ â€™ 1

ggplot(data2, aes(x = variable, y = value, color = variable, fill = variable)) +

geom_violin(alpha = 0.2) +

theme_bw()

ggplot(data2, aes(x = variable, y = value, color = variable, fill = variable)) +

geom_violin(alpha = 0.2) +

geom_point(position = position_jitter(0.3)) +

theme_bw()

ggplot(data2, aes(x = variable, y = value, color = variable, fill = variable)) +

geom_boxplot(alpha = 0.2) +

geom_point(position = position_jitter(0.3)) +

theme_bw()

ggplot(data2, aes(x = variable, y = value, color = variable, fill = variable)) +

geom_violin(alpha = 0.1) +

geom_boxplot(alpha = 0.1) +

geom_point(position = position_jitter(0.3)) +

theme_bw()

ggplot() +

geom_violin(data = data2, aes(x = variable, y = value, color = variable, fill = variable), alpha = 0.1) +

geom_errorbar(data = data3, aes(x = variable, ymin=mean-sd, ymax=mean+sd), width = 0.2)
