# Supplementary material for: High expression of RRM2 mediated by non-coding RNAs correlates with poor prognosis and tumor immune infiltration of hepatocellular carcinoma
Source: Front Med (Lausanne). 2022 Jul 14;9:833301. doi: 10.3389/fmed.2022.833301 (PMC9330188; doi:10.3389/fmed.2022.833301)
Supplement: Supplementary file 2 [file Data_Sheet_2.docx]

Supplementary Material

# Supplementary Figures


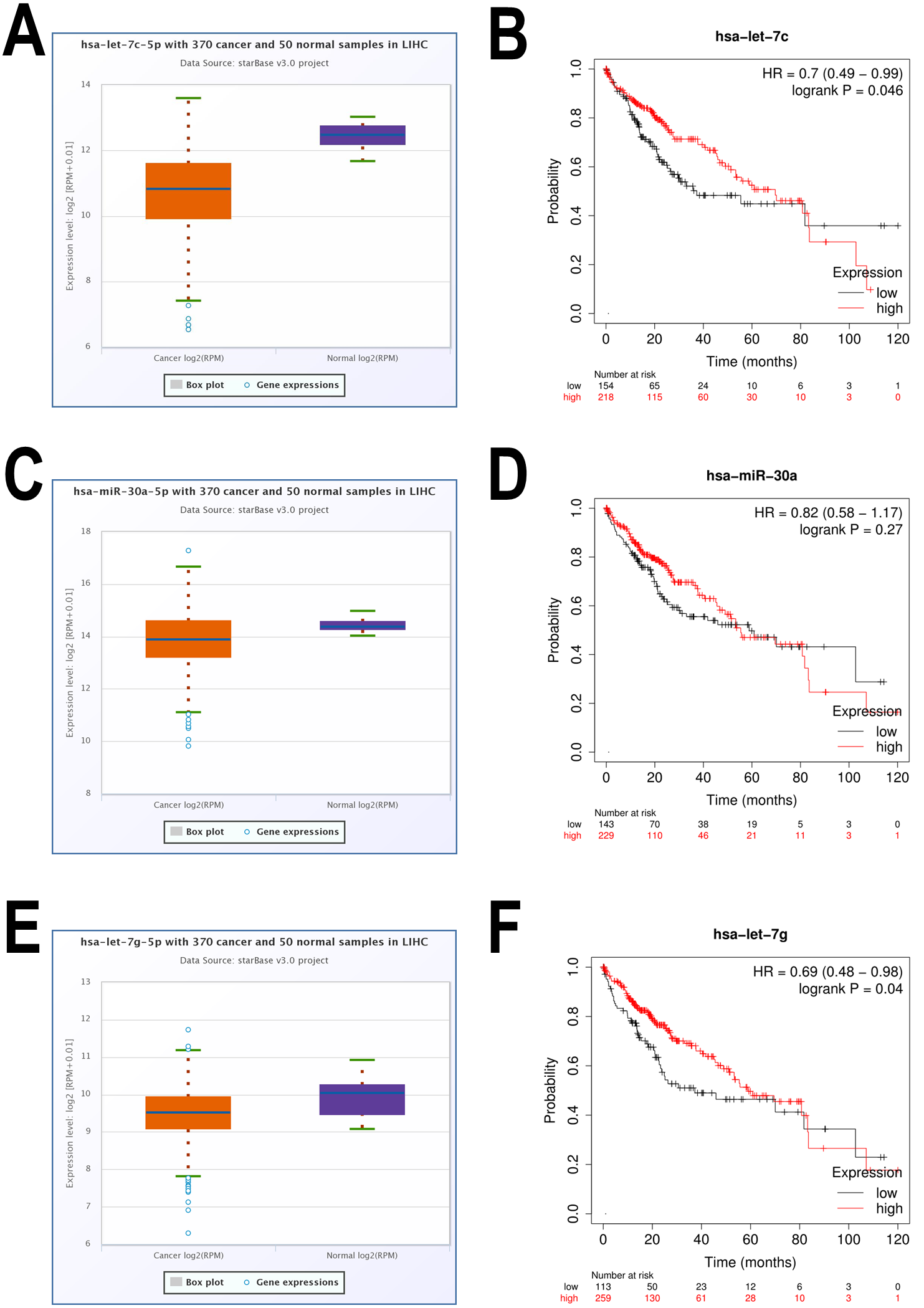


**Supplementary Figure 1.** The expression and prognostic value of candidate miRNAs in HCC. (A-F) The expression and prognostic value of let-7c-5p (A and B), miR-30a-5p (C and D) and let-7g-5p (E and F) in HCC were detected by starBase and Kaplan Meier plotter.


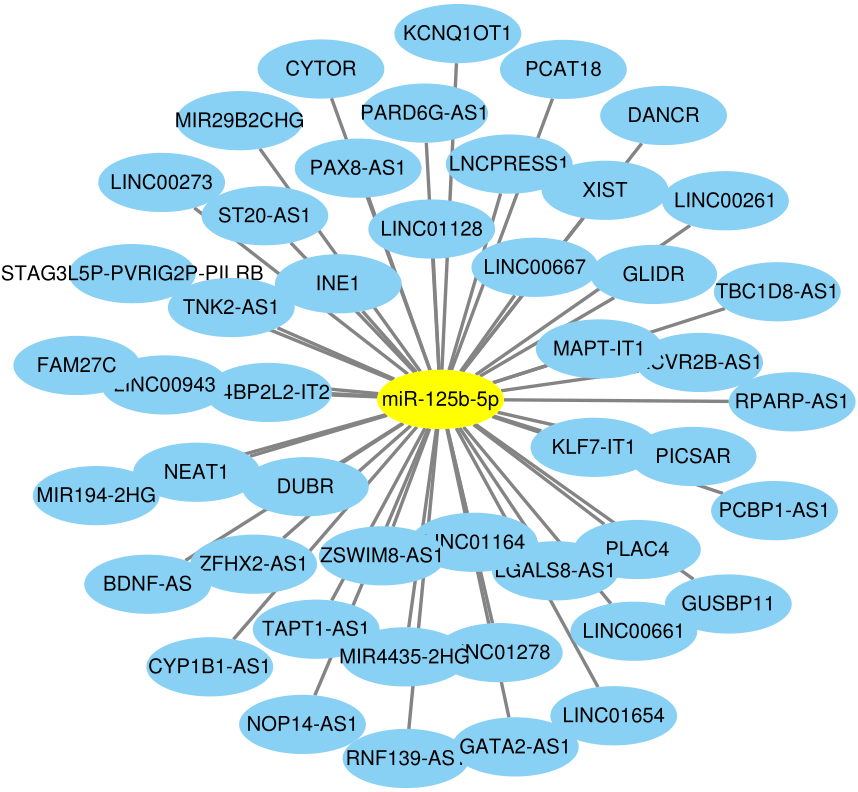


**Supplementary Figure 2.** The lnRNAs - miR-125b-5p prediction network produced by Cytoscape
